# Mental fatigue and impaired cognitive function after an acquired brain injury

**DOI:** 10.1002/brb3.1056

**Published:** 2018-06-29

**Authors:** Axel Jonasson, Christopher Levin, Marielle Renfors, Sara Strandberg, Birgitta Johansson

**Affiliations:** ^1^ Neuropsykiatrin Mölndal Sweden; ^2^ Resurscentrum Kärnhuset Halmstad Sweden; ^3^ Rehabiliteringskliniken Växjö Sweden; ^4^ Rehabiliteringsmottagningen Länssjukhuset Kalmar Sweden; ^5^ Institute of Neuroscience and Physiology University of Gothenburg Gothenburg Sweden

**Keywords:** acquired brain injury, cognitive performance, mental fatigue

## Abstract

**Objective:**

Mental fatigue is a common subjective symptom following an acquired brain injury. In many cases, this is long‐lasting with a considerable negative impact on work, studies, social activities, and quality of life. No objective test for mental fatigue exists today. The aim of this study was to investigate whether mental fatigue can be objectively measured.

**Materials and Methods:**

This study included 32 controls with no mental fatigue and 42 well‐rehabilitated participants who suffered from long‐term mental fatigue following stroke, traumatic brain injury, encephalitis or meningitis, and late effects after brain tumor. Attention, processing speed and working memory were assessed using a test and retest design following a demanding reading exercise.

**Results:**

Significant interactions were found for tests measuring processing speed, working memory, and attention. The control group improved at the second test, while those who suffered from mental fatigue did not.

**Conclusions:**

This study indicates impaired cognitive performance over time after cognitive activity for individuals suffering from mental fatigue after an acquired brain injury.

## 1 INTRODUCTION

Mental fatigue is a common symptom following traumatic brain injury (TBI) (Cantor, Gordon, & Gumber, 2013), stroke (Staub & Bogousslavsky, 2001), meningitis or encephalitis (Veje et al., 2016), and neurological diseases (Penner & Paul, 2017). In the case of long‐lasting mental fatigue, even once a person has recovered, they still may have difficulty returning to work and pursuing social activities. Objective measurement of fatigue remains challenging, but self‐perceived fatigue can be assessed with questionnaire‐based reporting scales.

Fatigue has been defined as “The awareness of a decreased capacity for physical and/or mental activity due to an imbalance in the availability, utilization, and/or restoration of resources needed to perform activity.” (Aaronson et al., 1999) This fits with the related definition of mental fatigue with an inability to repeatedly sustain cognitive performance and the need for a long recovery time after exertion (Johansson and Rönnbäck, 2014a).

Fatigue after an acquired brain injury has been explained by the coping hypothesis, which suggests that the brain needs to work harder to compensate for impairments to cognitive functions such as attention and processing speed, which in turn results in fatigue (van Zomeren & van den Burg, 1985). The coping hypothesis has been supported by several authors (Belmont, Agar, Hugeron, Gallais, & Azouvi, 2006; Ziino and Ponsford, 2006a,b; Belmont, Agar, & Azouvi, 2009). Azouvi and co‐authors proposed that mentally‐tiring activities after brain injury are attributable to reduced resources and that patients who have sustained a brain injury also describe mental activity as more energy demanding than healthy people (Azouvi et al., 2004). In an assessment of decreased cognitive function combined with mental fatigue, it has been proposed that subjective fatigue after TBI or mild TBI correlates with poor performance in attention tests and reduced processing speed (Ziino and Ponsford, 2006a,b; Belmont et al., 2009; Azouvi et al., 2004; Ashman et al., 2008; Johansson, Berglund, & Rönnbäck, 2009; Park, Moscovich, & Robertson, 1999; Ponsford, Cameron, Fitzgerald, Grant, & Mikocka‐Walus, 2011). A group of individuals who had sustained a TBI performed more slowly on a complex attention test, made more errors, and reported a higher level of subjective fatigue (Ziino and Ponsford, 2006a). Their performance was slower, but remained on the same level during a vigilance test (Ziino and Ponsford, 2006b). Moreover, a simultaneous load on working memory requiring total control of the situation was more tiring for TBI subjects than an automatic activity (Park et al., 1999). After a severe TBI, subjects showed an increase in reaction time during a dual‐task activity, and they reported an increase in subjective mental effort (Azouvi et al., 2004). Patients with self‐reported fatigue after a severe TBI performed less well than controls on a selective attention test. This finding also correlated with fatigue and mental effort (Belmont et al., 2009). Ponsford and co‐authors reported a study on post‐mild TBI but well‐rehabilitated patients. These patients performed less well than controls on a visual memory test and reported problems with fatigue after 3 months (Ponsford et al., 2011).

Traditionally, neuropsychological tests measure cognitive function at a single time point. From the reports above, differences at the group level can be found. However, the difference at group level is not applicable in the clinic because of significant variation between patients, and it is difficult to differentiate between results due to fatigue and results due to the injury per se. However, the mental fatigue quantified by measuring changes in cognitive performance over time is rarely reported, but this is what patients often report. Few studies using repetition of cognitive tests indicate the level of difficulty in improving cognitive performance if perceived fatigue is present, whereas improvement can be achieved for healthy controls. Repetition of a computerized test with simultaneous demand on divided attention and working memory over a short period of 4 minutes showed an interaction effect, with the controls improving their speed, while this was not found for those suffering from mental fatigue after a mild TBI (Johansson & Rönnbäck, 2015). Practice increased the response speed over time (test period in total 3.5 hr) for the controls, while this was not the case for those suffering from fatigue after a TBI (Ashman et al., 2008). Furthermore, a diurnal decline in cognitive function was reported for multiple sclerosis and stroke patients suffering from fatigue compared with controls (Claros‐Salinas et al., 2010).

The intention of this study was to investigate whether it is possible to measure mental fatigue using a test and retest design. The hypothesis is that those who had sustained an acquired brain injury and who suffer from long‐term mental fatigue will fail to improve on a cognitive task when this is repeated after a demanding activity, whereas the participants in the control group are able to improve.

## 2 METHODS

### 2.1 Participants

Forty‐two participants with an acquired brain injury suffering from mental fatigue were recruited from two clinics in southeast Sweden specializing in acquired brain injury rehabilitation and from the Institute of Neuroscience, at The Sahlgrenska Academy, University of Gothenburg, where mental fatigue studies are performed. Twenty participants had suffered a TBI or mild TBI, 13 a stroke, 6 encephalitis/meningitis, and 3 treated brain tumor. Five participants were receiving medication for their mental fatigue after a TBI, using short‐acting methylphenidate (a central nervous system stimulant), but they had not taken any methylphenidate 24 hr before the test. The half‐life of short‐acting methylphenidate is 2–3 hr. All mental fatigue participants had recovered well, were independent in their daily lives, with the exception of their prolonged mental fatigue. Exclusion criteria were drug and alcohol misuse and psychiatric disorders. The control group included 32 participants. These participants had the capacity to work full‐time and had not experienced a previous acquired brain injury, nor any drug or alcohol misuse or psychiatric disorder, and were matched to age, gender, and education. The control group was recruited by personal requests. All participation was voluntary, and no reward was given for participating. Written informed consent was provided to participants, and the study was approved by the regional ethical review board in Gothenburg.

### 2.2 Measures

The Mental Fatigue Scale (MFS) was used for assessment of mental fatigue (Johansson and Rönnbäck, 2014b; Johansson, Starmark, Berglund, Rödholm, & Rönnbäck, 2010) and is based on the definition of mental fatigue suggested by Johansson and Rönnbäck (Johansson and Rönnbäck, 2014a). The MFS is a multidimensional questionnaire comprising 15 questions with a cutoff score of 10.5. Typical MFS items include impaired mental fatigue, long recovery time, and concentration difficulties. Associates symptoms are lack of initiative, memory problems, slowness of thinking, sensitivity to stress, increased tendency to become emotional, irritability, sensitivity to light and noise, and decreased or increased sleep (Johansson and Rönnbäck, 2014b).

**Figure 1:**
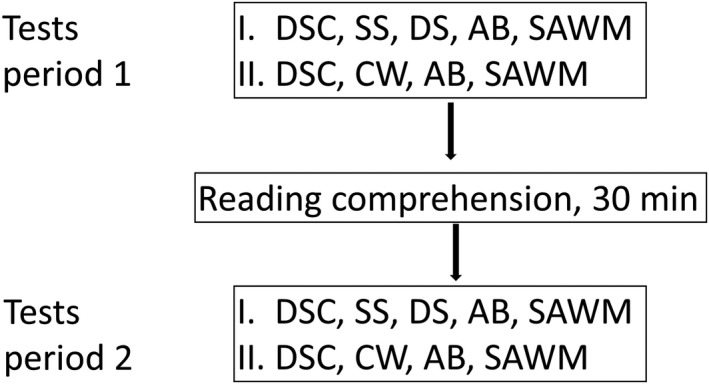
Study design. Participants repeated the same tests, except Symbol Search and Digit Span which were added in test battery I and Color Word, added in test battery II

The test for cognitive performance was divided into pre‐ and posttest sections and between these a reading comprehension test, lasting for 30 minutes. Two similar neuropsychological test and retest batteries were used for the assessment (Figure 1). Both test batteries included a) Digit Symbol Coding (DSC, WASI‐IV) (Wechsler, 2010), b) Attentional Blink test (AB) (Slagter et al., 2007; Dux & Marois, 2009), and c) a computerized test combining divided attention and working memory simultaneously (SAWM) (Johansson & Rönnbäck, 2015). Test battery I also included Digit Span (DS) and Symbol search (SS)(WAIS‐IV) (Wechsler, 2010) and test battery II included D‐KEFS Color Word (CW) (Delis, Kaplan, & Kramer, 2001). The total assessment period, including reading comprehension, was approximately 1 hr 45 min.

The selection of tests was based on previous studies reporting information processing speed, attention, and working memory as the cognitive functions most susceptible after an acquired brain injury (Mathias, Beall, & Bigler, 2004), and impaired performance also related to fatigue after a brain injury (Ziino and Ponsford, 2006a; Ashman et al., 2008; Johansson et al., 2009). The interest in this study was to study mental fatigue, comparing pre‐ and posttest results between the mental fatigue group and the controls. With a slight difference in test batteries, we were also able to include more tests in total, without increasing the total assessment time.

Digit span consists of three subtests: DS forward, DS backward, and DS sequencing which measures attention and working memory. DS forward activates attention, encoding, and auditory processing. DS backward and sequencing activate working memory and mental manipulation (Wechsler, 2010). SS and DSC are subtests within the Processing Speed Index in WAIS‐IV (Wechsler, 2010), relating to attention, short‐term visual memory, speed of mental and psychomotor operation, and visual discrimination. CW from Delis‐Kaplan Executive Function System (D‐KEFS) (Delis et al., 2001) is based on the Stroop procedure (Stroop, 1935), which primarily examines the ability to inhibit an overlearned verbal response, that is reading the word and not naming the ink color the words are printed in. The test also measures basic speed of naming the color of squares and reading the printed word for the color.

In this study, the AB task is based on the description from Slagter et al. (Slagter et al., 2007). In this computer task, the individual is shown a rapid stream of events, specifically letters, with two targets (digits, Target 1 = T1 and Target 2 = T2) embedded in the rapid stream of letters. We presented 17 uppercase letters and two digits (15‐mm high) in black on a light gray background in random order in the center of the computer screen. Each trial started with the presentation of a 1780‐ms fixation cross, followed by the rapid stream of letters which were randomly selected (except for B, I, O, Q, Å, Ä, Ö). Two randomly selected digits between 2 and 9 were embedded in the stream of letters. Each letter or digit was presented for 50 ms and was followed by a blank screen for 34 ms. The T1 was always presented as the fifth item in the letter stream. The temporal distance between digits T1 and T2 was 252 ms (short), 504 ms (medium), or 756 ms (long), and this distance was randomly drawn. There were 10 trials for each temporal distance for a total of 30 trials. The participants were informed that two digits would always be presented, and they were asked to report these digits after each trial at their own pace. If they were unsure, they were asked to guess. After a practice block of eight trials, they performed one run consisting of 30 trials. The primary measure of interest was the percentage of correct T2 reports from the trials in which T1 was accurately identified.

The computerized test includes simultaneous divided attention and working memory (SAWM) (Johansson & Rönnbäck, 2015). The test measures speed of mouse clicks in four squares, located in each corner of a larger square on the computer screen, and is performed in a clockwise order. At the same time, the subject was asked to count how many instances of a specific digit were shown (seen in the square to the upper right). A new digit, between zero and nine was randomly chosen for each run. The digits to be counted were randomly displayed in the upper square to the left for 1 second. After the 30 seconds, the subject was asked to report how many of the specific digits he/she had seen. The number of clicks was simultaneously recorded. Each session lasted for 30 seconds and was repeated five times. All participants worked through one repetition to ensure that they understood the task. This first repetition was not included in the analysis.

The reading comprehension test used was a subtest of the Swedish Scholastic Aptitude Test (SweSAT), called LÄS, originally given during the SweSAT autumn semester 2010 (block number 5, subtest number 9). It contained five different texts each covering one to one and a half A4‐pages. Every text was followed by four questions relating to the text, with four alternative answers provided for every question (A–D). The questions demand the ability to notice details, as well as the capacity to conclude from the content of the complete text. The test was assumed to be demanding as reading is commonly reported as strenuous and mental fatigue sufferers read more slowly compared with healthy people (Johansson et al., 2009).

### 2.3 Data analysis

The data were analyzed using a 2 × 2 mixed design repeated‐measurement analysis of variance in order to investigate interaction between group and time (group × time). As age differed significantly between the groups, age was controlled for in all the analyses (covariate analysis of variance, ANCOVA). Sphericity and internal variance were checked for. Paired *t*‐test for each group was used for comparison of pre‐ and posttest results when interaction effect was detected. For comparison between the groups, analysis of variance (ANOVA), *t* test, and Chi‐square test were used. Correlations were measured with Pearson's correlation. SPSS http://scicrunch.org/resolver/SCR_002865 21.0 was used for statistical calculations.

## 3 RESULTS

The groups differed significantly in age, with the control group being slightly younger (Table 1). No significant difference between the groups was found for numbers of males and females or for level of education (Table 1). The mental fatigue group scored significantly higher on the MFS than the control group (Table 1). The rating on MFS for the different clinical acquired brain injury diseases was on a similar level, and no significant difference between them was found (*p* = 0.497, mean MFS; TBI 20.9, Stroke 18.5, encephalitis/meningitis 18.7, brain tumor 19.7). No significant difference between the scores on the MFS for females and males was detected. Females from the mental fatigue group scored 20.4 (mean) and males 18.5 (*t*‐test, *p* = 0.230) and control females 4.0 and males 3.0 (*t*‐test, *p* = 0.236). Age did not correlate with MFS score (*r* = −0.137, *p* = 0.385). Median time since acquired brain injury was 36 months and the range was 2‐528 months. There was no significant correlation between time since injury and MFS score (*r* = 0.183, *p* = 0.246).

**Table 1:** Participant characteristics. Statistical comparisons between the groups, with the mean, standard deviation in parentheses, and frequencies presented

	Fatigue group (42)	Control group (32)	*t*/*F*‐value	*p*‐value
Age, years (*t*‐test^a^)	45.0 (11.6)	38.8 (12.5)	2.193	0.032
Sex, females/males (Chi‐square)	28/14	15/17		0.087
MFS (*t*‐test^b^)	19.7 (4.7)	3.4 (2.3)	19.568	<0.001
Time since acquired brain injury, median	36 months	—		
Level of education (Chi‐square)				
Elementary school	6	1		0.243
High school	11	8		
University	25	23		

^a^Equal variances, ^b^equal variances not assumed.

**Figure 2:**
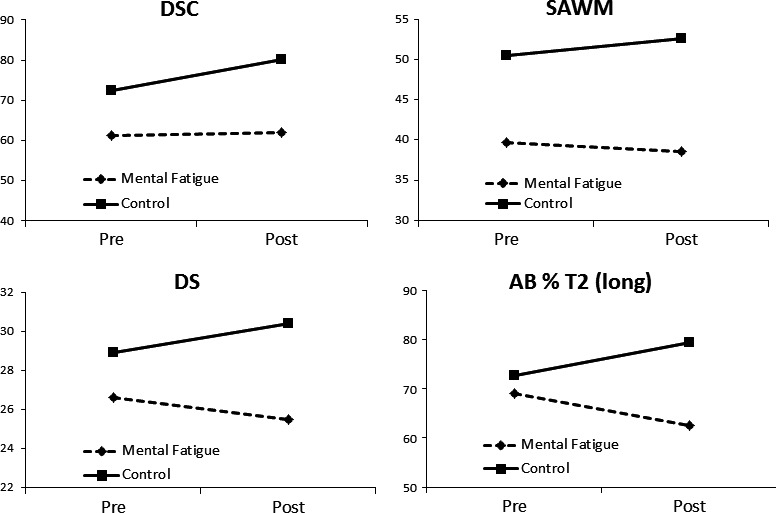
Cognitive tests with a significant interaction (time × group) are shown. The controls improved, while those who suffered from mental fatigue after an acquired brain injury did not or they performed on a lower level when the tests were repeated after 1 hr

All cognitive tests were controlled for age. With the repeated‐measure analysis comparing pre‐ and posttest and group differences, significant interaction effects were found for a) DSC, b) DS total score and the subtest sequencing, c) SAWM speed, and d) AB with the longest duration between the two digits. The results are shown in Tables 2 and 3 and Figure 2. With a paired *t*‐test, significant improvement for DSC and the SAWM speed were found for the control, while no significant change was detected for the mental fatigue group (paired *t*‐test, DSC *p* > 0.001 and SAWM 0.005).

**Table 2:** Neuropsychological raw scores for the first (1) and second (2) test periods for each group are presented with mean and standard deviation (in parenthesis)

Test	ABI‐MF Group	Control Group
DSC 1 and 2	61.3 (16.9); 62.0 (19.1)	72.5 (13.6); 80.1(15.4)
SS 1 and 2	29.0 (7.8); 30.0 (9.6)	35.9 (8.0); 40.1 (8.2)
DS total 1 and 2	26.6 (4.5); 25.5 (5.2)	28.9 (4.1); 30.4 (4.9)
DS forward 1 and 2	8.7 (1.5); 8.7 (1.6)	9.6 (1.9); 10.4 (2.2)
DS backwards 1 and 2	9.3 (2.4); 8.6 (2.9)	10.0 (1.8); 9.9 (2.8)
DS sequencing 1 and 2	8.6 (1.1); 8.2 (1.7)	9.2 (2.0); 10.2 (1.6)
CW (naming colors) 1 and 2	33.8 (11.4); 36.7 (8.7)	27.0 (4.9); 26.1 (6.1)
CW (reading) 1 and 2	25.9 (6.2); 28.8 (6.4)	20.9 (3.4); 20.7 (5.6)
CW (interference) 1 and 2	63.3 (15.0); 65.1 (25.0)	47.1 (10.5); 43.7 (10.4)
SAWM speed 1 and 2	39.7 (10.6); 38.5 (10.7)	50.5 (12.8); 52.6 (13.0)
SAWM error 1 and 2	0.48 (0.98); 0.44 (1.19)	0.12 (0.39); 0.15 (0.49)
AB % T2 (short) 1 and 2	40.3 (30.7); 43.1 (28.9)	54.6 (29.8); 53.3 (27.1)
AB % T2 (medium) 1 and 2	67.5 (25.8); 58.9 (29.6)	78.3 (23.6); 77.3 (25.3)
AB % T2 (long) 1 and 2	69.1 (25.0); 62.5 (25.2)	72.8 (23.0); 79.5 (21.4)

Note.

ABI‐MF: acquired brain injury group suffering from mental fatigue; DSC: Digit Symbol Coding; SS: Symbol Search; DS: Digit Span; CW: Color word; SAWM: Computer test with simultaneous measure of speed, attention, and working memory; AB: Attentional Blink test.

**Table 3:** Results from the repeated analysis of variance, all tests controlling for age

Test	N ABI‐MF/C	Interaction, time × group	Group difference
DSC	42/32	*F* = 9.289, *p* = 0.003, *ƞ* ^2^ = 0.116	*F* = 10.888, *p* = 0.002, *ƞ* ^2^ = 0.133
SS	19/17	*F* = 1.529, *p* = 0.225, *ƞ* ^2^ = 0.044	*F* = 8.104, *p* = 0.008, *ƞ* ^2^ = 0.197
DS total	19/17	*F* = 5.418, *p* = 0.026, *ƞ* ^2^ = 0.141	*F* = 5.342, *p* = 0.027, *ƞ* ^2^ = 0.139
DS forward	19/17	*F* = 1.109, *p* = 0.300, *ƞ* ^2^ = 0.033	*F* = 4.577, *p* = 0.040, *ƞ* ^2^ = 0122
DS backwards	19/17	*F* = 0.470, *p* = 0.498, *ƞ* ^2^ = 0.014	*F* = 1.698, *p* = 0.202, *ƞ* ^2^ = 0.049
DS sequencing	19/17	*F* = 6.291, *p* = 0.017, *ƞ* ^2^ = 0.160	*F* = 6.995, *p* = 0.012, *ƞ* ^2^ = 0.175
CW (naming)	23/15	*F* = 2.277, *p* = 0.140, *ƞ* ^2^ = 0.061	*F* = 12.449, *p* = 0.001, *ƞ* ^2^ = 0.262
CW (reading)	23/15	*F* = 3.820, *p* = 0.059, *ƞ* ^2^ = 0.098	*F* = 16.980, *p* < 0.001, *ƞ* ^2^ = 0.327
CW (interference)	23/15	*F* = 1.607, *p* = 0.213, *ƞ* ^2^ = 0.044	*F* = 12.795, *p* = 0.001, *ƞ* ^2^ = 0.268
SAWM, speed	42/32	*F* = 12.280, *p* = 0.001, *ƞ* ^2^ = 0.147	*F* = 15.397, *p* < 0.001, *ƞ* ^2^ = 0.178
SAWM, error	42/32	*F* = 0.276, *p* = 0.601, *ƞ* ^2^ = 0.004	*F* = 4.039, *p* = 0.048, *ƞ* ^2^ = 0.054
AB % T2 (short)	42/32	*F* = 0.046, *p* = 0.832, *ƞ* ^2^ = 0.001	*F* = 3.939, *p* = 0.051, *ƞ* ^2^ = 0.053
AB % T2 (medium)	42/32	*F* = 1.574, *p* = 0.214, *ƞ* ^2^ = 0.022	*F* = 5.584, *p* = 0.021, *ƞ* ^2^ = 0.073
AB % T2 (long)	42/32	*F* = 7.211, *p* = 0.009, *ƞ* ^2^ = 0.092	*F* = 3.072, *p* = 0.084, *ƞ* ^2^ = 0.041

Notes.

The interaction factor is time × group and the last column between group comparisons. *F*‐ and *p*‐value, and effect size (partial eta squared, *ƞ* ^2^) are presented. Main significant time effect was only detected for Digit Symbol Coding (*p* < 0.01) and Symbol Search (*p* = 0.01).

N is the number of individuals for each test and group, ABI‐MF: Acquired brain injury group suffering from mental fatigue; C: controls; DSC: Digit Symbol Coding; SS: Symbol Search; DS: Digit Span; CW: Color Word; SAWM: Computer test with simultaneous measure of speed, attention, and working memory; AB: Attentional Blink test.

In almost all cognitive tests included in this study, except DS backward and AB short and long, the control group performed significantly better than the mental fatigue group (Tables 2 and 3). The control group also performed significantly better on the Reading comprehension task (*p* = 0.001, *t*‐test equal variances not assumed), with more correct answers 10.6 (*SD* 3.2) than the mental fatigue group (7.5, *SD* 4.5).

## 4 DISCUSSION

While fatigue is a well‐established common symptom of neurological diseases, it remains understudied and is poorly understood (Penner & Paul, 2017). Typically, mental exhaustion becomes pronounced during sensory stimulation or when cognitive tasks have to be performed for extended periods. Today, fatigue is assessed with subjective scales, and it has been proven difficult to measure fatigue objectively. The fatigue scales take into consideration the perceived fatigue and is related to the total activity scores as well as taking into account the demands of daily mental activities. Traditionally, neuropsychological tests measure cognitive function at a single time point and do not consider changes in cognitive performance over time. However, it is the ability to repeatedly perform tasks that is critical for people suffering from mental fatigue.

The main focus in this study was to explore performance in cognitive tests which were repeated and interleaved with a demanding reading task. The repeated analysis comparing pre‐ and posttest showed significant interaction effects for tests measuring processing speed (DSC, SAWM speed), working memory (DS total score and the subtest sequencing), and attention (AB). The control group improved on DSC and SAWM, while the mental fatigue group remained on a similar level or showed a tendency to decline. From two other studies, similar results have been reported. Ashman et al. (Ashman et al., 2008). reported improved response speed for controls from pre‐ to posttest within a single assessment period, while no improvement was found for those suffering from fatigue after a TBI. Johansson & Rönnbäck, (2015) reported improvement in speed for controls when the SAWM test was repeated, while sufferers from mental fatigue after a mild TBI did not. These studies indicate that people suffering from mental fatigue after an acquired brain injury find it challenging to repeat cognitive tasks, whereas improvement can be achieved for healthy controls.

The control group performed significantly better on all cognitive tests included in this study, compared with those who suffered from mental fatigue after an acquired brain injury. This finding is in accordance with other studies proposing that subjective fatigue after TBI and stroke correlates with poor performance on single tests for attention and processing speed (Ziino and Ponsford, 2006a,b; Belmont et al., 2009; Azouvi et al., 2004; Ashman et al., 2008; Johansson et al., 2009; Park et al., 1999; Ponsford et al., 2011; Johansson & Rönnbäck, 2012). However, it is difficult to determine whether the impairment in cognitive function is due to fatigue or the injury per se or a combination of the two. From the results in the study, we suggest the interaction effect or, more specifically, the lack of improvement from pre‐ to posttest to be attributable to individual fatigue. As we did not include a group with an acquired brain injury not suffering from mental fatigue, it is not possible to differentiate between fatigue and brain injury.

### 4.1 Limitations

Participants in this study suffered all from mental fatigue after stroke, TBI, meningitis/encephalitis, or brain tumor. While these can be regarded as a heterogeneous group of patients, they all rated their mental fatigue on a similar level and were, thus, treated as one group. The broad range of time elapsed since injury did not correlate with MFS. The age differed significantly between clinical and control groups. However, we controlled for age in all cognitive tests and we did not detect any correlation to age and MFS in this study.

In conclusion, this study indicates impaired cognitive performance during an extended test session for those suffering from mental fatigue after an acquired brain injury. This lack of ability to repeatedly perform tasks without mental exhaustion is critical for daily living, but is rarely studied. However, repeated cognitive testing is a promising method for the objective measurement of mental fatigue. No single, traditional neuropsychological test captures the endurance of cognitive performance. Mental fatigue is challenging for both healthcare professionals and patients, and it is an important research field, as mental fatigue affects people tremendously in their struggle to return to work and also in their efforts to find a balanced workload which is sustainable over a longer period.

## CONFLICT OF INTEREST

The authors have no competing interest to declare.

## References

[brb31056-bib-0001] Aaronson, L. S. , Teel, C. S. , Cassmeyer, V. , Neuberger, G. B. , Pallikkathayil, L. , Pierce, J. , … Wingate, A. (1999). Defining and measuring fatigue. Image ‐ The Journal of Nursing Scholarship, 31(1), 45–50. 10.1111/j.1547-5069.1999.tb00420.x 10081212

[brb31056-bib-0002] Ashman, T. A. , Cantor, J. B. , Gordon, W. A. , Spielman, L. , Egan, M. , Ginsberg, A. , … Flanagan, S. (2008). Objective measurement of fatigue following traumatic brain injury. Journal of Head Trauma Rehabilitation, 23(1), 33–40. 10.1097/01.HTR.0000308719.70288.22 18219233

[brb31056-bib-0003] Azouvi, P. , Couillet, J. , Leclercq, M. , Martin, Y. , Asloun, S. , & Rousseaux, M. (2004). Divided attention and mental effort after severe traumatic brain injury. Neuropsychologia, 42, 1260–1268. 10.1016/j.neuropsychologia.2004.01.001 15178177

[brb31056-bib-0004] Belmont, A. , Agar, N. , & Azouvi, P. (2009). Subjective fatigue, mental effort, and attention dificits after severe traumatic brain injury. Neurorehabilitation and Neural Repair, 23(9), 939–944. 10.1177/1545968309340327 19574545

[brb31056-bib-0005] Belmont, A. , Agar, N. , Hugeron, C. , Gallais, B. , & Azouvi, P. (2006). Fatigue and traumatic brain injury. Annales de readaptation et de medecine physique, 49, 283–288. 10.1016/j.annrmp.2006.04.017 16716438

[brb31056-bib-0006] Cantor, J. B. , Gordon, W. , & Gumber, S. (2013). What is post TBI fatigue? NeuroRehabilitation, 32, 875–883. 23867414 10.3233/NRE-130912

[brb31056-bib-0007] Claros‐Salinas, D. , Bratzke, D. , Greitemann, G. , Nickisch, N. , Ochs, L. , & Schröter, H. (2010). Fatigue‐related diurnal variations of cognitive performance in multiple sclerosis and stroke patients. Journal of the Neurological Sciences, 295(1–2), 75–81. 10.1016/j.jns.2010.04.018 20510427

[brb31056-bib-0008] Delis, D. C. , Kaplan, E. , & Kramer, J. H. (2001). Delis‐kaplan executive function system – D‐KEFS. San Antonio, TX: The Psychological Corporation.

[brb31056-bib-0009] Dux, P. E. , & Marois, R. (2009). The attentional blink: A review of data and theory. Attention Perception and Psychophysics, 71(8), 1683–1700. 10.3758/APP.71.8.1683 PMC2915904 19933555

[brb31056-bib-0010] Johansson, B. , Berglund, P. , & Rönnbäck, L. (2009). Mental fatigue and impaired information processing after mild and moderate traumatic brain injury. Brain Injury, 23(13–14), 1027–1040. 10.3109/02699050903421099 19909051

[brb31056-bib-0011] Johansson, B. , & Rönnbäck, L. (2012). Mental fatigue and cognitive impairment after an almost neurological recovered stroke. International Scholarly Research Network Psychiatry, 2012(Article ID 686425), 7. 10.5402/2012/686425 PMC3658493 23738208

[brb31056-bib-0012] Johansson, B. , & Rönnbäck, L. (2014a). Long‐lasting mental fatigue after traumatic brain injury – A major problem most often neglected diagnostic criteria, assessment, relation to emotional and cognitive problems, cellular background, and aspects on treatment In Sadaka F. (Ed.), Traumatic brain injury. Rijeka, Croatia: INTECH.

[brb31056-bib-0013] Johansson, B. , & Rönnbäck, L. (2014b). Evaluation of the mental fatigue scale and its relation to cognitive and emotional functioning after traumatic brain injury or stroke. International Journal of Physical Medicine and Rehabilitation, 2, 182.

[brb31056-bib-0014] Johansson, B. , & Rönnbäck, L. (2015). Novel computer tests for identification of mental fatigue after traumatic brain injury. NeuroRehabilitation, 36(2), 195–202. 25882202 10.3233/NRE-151207

[brb31056-bib-0015] Johansson, B. , Starmark, A. , Berglund, P. , Rödholm, M. , & Rönnbäck, L. (2010). A self‐assessment questionnaire for mental fatigue and related symptoms after neurological disorders and injuries. Brain Injury, 24(1), 2–12. 10.3109/02699050903452961 20001478

[brb31056-bib-0016] Mathias, J. L. , Beall, J. A. , & Bigler, E. D. (2004). Neuropsychological and information processing deficits following mild traumatic brain injury. Journal of the International Neuropsychological Society, 10, 286–297. 15012849 10.1017/S1355617704102117

[brb31056-bib-0017] Park, N. W. , Moscovich, M. , & Robertson, I. H. (1999). Divided attention impairments after traumatic brain injury. Neuropsychologia, 37(10), 1119–1133. 10.1016/S0028-3932(99)00034-2 10509834

[brb31056-bib-0018] Penner, I. K. , & Paul, A. (2017). Fatgue as a symptom or comorbidity of neurological diseases. Nature Reviews Neurology, 13, 662–675. 10.1038/nrneurol.2017.117 29027539

[brb31056-bib-0019] Ponsford, J. , Cameron, P. , Fitzgerald, M. , Grant, M. , & Mikocka‐Walus, A. (2011). Long‐term outcomes after uncomplicated mild traumatic brain injury: A comparison with trauma controls. Journal of Neurotrauma, 28, 937–946. 10.1089/neu.2010.1516 21410321

[brb31056-bib-0020] Slagter, H. A. , Lutz, A. , Greischar, L. L. , Francis, A. D. , Nieuwenhuis, S. , Davis, J. M. , & Davidson, R. J. (2007). Mental training affects distribution of limited brain resources. Public Library of Science (PLOS) Biology, 5(6), e138. 10.1371/journal.pbio.0050138 PMC1865565 17488185

[brb31056-bib-0021] Staub, F. , & Bogousslavsky, J. (2001). Fatigue after stroke: A major but neglected issue. Cerebrovascular Diseases, 12, 75–81. 10.1159/000047685 11490100

[brb31056-bib-0022] Stroop, J. R. (1935). Studies of interference in serial verbal reactions. Journal of Experimental Psychology, 18(6), 643–662. 10.1037/h0054651

[brb31056-bib-0023] Veje, M. , Nolskog, P. , Petzold, M. , Bergström, T. , Lindén, T. , Peker, Y. , & Studahl, M. (2016). Tick‐Borne Encephalitis sequelae at long‐term follow‐up: A self‐reported case‐control study. Acta Neurologica Scandinavica, 134(6), 434–441. 10.1111/ane.12561 26810689

[brb31056-bib-0024] Wechsler, D. (2010). Wechsler adult intelligence scale – fourth edition, Swedish version. Stockholm, Sweden: Pearson Assessment.

[brb31056-bib-0025] Ziino, C. , & Ponsford, J. (2006a). Selective attention deficits and subjective fatigue following traumatic brain injury. Neuropsychology, 20, 383–390. 10.1037/0894-4105.20.3.383 16719631

[brb31056-bib-0026] Ziino, C. , & Ponsford, J. (2006b). Vigilance and fatigue following traumatic brain injury. Journal of the International Neuropsychological Society, 12(1), 100–110. 16433949 10.1017/S1355617706060139

[brb31056-bib-0027] van Zomeren, A. H. , & van den Burg, W. (1985). Residual complaints of patients two years after severe head injury. Journal of Neurology, Neurosurgery, and Psychiatry, 48(1), 21–28. 10.1136/jnnp.48.1.21 PMC1028178 3973618

